# Power efficient refined seizure prediction algorithm based on an enhanced benchmarking

**DOI:** 10.1038/s41598-021-02798-8

**Published:** 2021-12-06

**Authors:** Ziyu Wang, Jie Yang, Hemmings Wu, Junming Zhu, Mohamad Sawan

**Affiliations:** 1 Cutting-Edge Net of Biomedical Research and INnovation (CenBRAIN), School of Engineering, Westlake University, Hangzhou, Zhejiang China; 2 Department of Neurosurgery, Second Affiliated Hospital, Zhejiang University School of Medicine, Hangzhou, Zhejiang China

**Keywords:** Biomedical engineering, Epilepsy, Data processing, Machine learning

## Abstract

Deep learning techniques have led to significant advancements in seizure prediction research. However, corresponding used benchmarks are not uniform in published results. Moreover, inappropriate training and evaluation processes used in various work create overfitted models, making prediction performance fluctuate or unreliable. In this study, we analyzed the various data preparation methods, dataset partition methods in related works, and explained the corresponding impacts to the prediction algorithms. Then we applied a robust processing procedure that considers the appropriate sampling parameters and the leave-one-out cross-validation method to avoid possible overfitting and provide prerequisites for ease benchmarking. Moreover, a deep learning architecture takes advantage of a one-dimension convolutional neural network and a bi-directional long short-term memory network is proposed for seizure prediction. The architecture achieves 77.6% accuracy, 82.7% sensitivity, and 72.4% specificity, and it outperforms the indicators of other prior-art works. The proposed model is also hardware friendly; it has 6.274 k parameters and requires only 12.825 M floating-point operations, which is advantageous for memory and power constrained device implementations.

## Introduction

**Figure 1:**
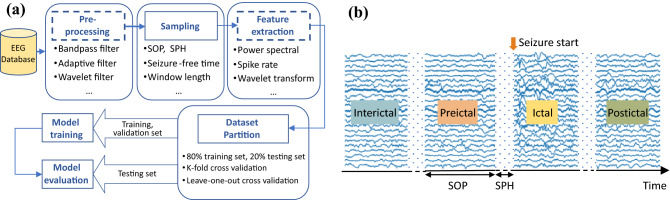
(**a**) The general process of machine learning or deep learning based seizure prediction task is: sampling, model training and model evaluation. The dashed boxes are not compulsory parts of deep learning based method. (**b**) Relationship of interictal, preictal, ictal and postictal. Ictal is during seizures, preictal is a period that precedes ictal, before SPH and within SOP. Postictal refers to the state after seizures, and interictal corresponds to normal stages.

Machine learning based seizure prediction has made great advancement over the past few years with the emerging of deep neural network^1–4^. In these studies, electroencephalography (EEG) signals are categorized into four types: ictal, preictal, postictal, and interictal, corresponding to seizure, before a seizure, after a seizure, and normal epileptic stages, respectively. Seizure prediction is regarded as a binary classification task to distinguish between preictal and interictal states^5–7^. A common process required to obtain such seizure prediction algorithms is summarized in Fig. 1a. The general steps are data pre-processing, sampling, feature extraction, model training, and model evaluation. Data pre-processing filters out noise and artifacts within EEG signals. Sampling refers to the process of collecting preictal and interictal samples from long-term EEG data. Feature extraction usually adopts time-frequency tools to extract useful features. Then a machine learning or deep learning model is trained to classify these samples, which will be further evaluated to ensure its generalization performance.

The machine learning methods usually refer to the traditional statistical methods. The EEG signals need to be pre-processed by filters to remove the noise and artifacts, such as bandpass filter, Kalman filter, and adaptive filter. Machine learning methods rely on hand-craft features such as spike rate^8^, mean phase coherence^9^, and power spectral density ratio^10^, which have been widely used in previous works. Moreover, by using Flourier transform, wavelet transform, and short-time Flourier transform, EEG signals could be analyzed from the frequency domain or time-frequency domain. However, these machine learning methods are difficult to apply clinically because these manually-engineered features are not only time-consuming but also affect the classification performance because some features are not informative enough for specific applications. With the development of deep learning, CNN has been proven to have built-in filtering and feature extraction abilities^11^. Thus, data pre-processing and feature extraction become optional steps for deep learning methods. Convolution layer, max-pooling layer, activation function, batch normalization, and dropout layer are the most common and basic operators, which could be composed into different networks. The recurrent neural network (RNN)^12^ and transformer^13^, which are widely used in natural language processing field, are also applied for seizure prediction in recent years^14, 15^.

Despite the growing popularity of machine learning based seizure prediction, the standards of sampling and model evaluation vary widely across different studies, making it difficult to compare different models directly and may also lead to overly optimistic results. Seizure prediction horizon (SPH) and seizure occur period (SOP) are two important parameters for sampling. As shown in Fig. 1b, the SOP is the interval where the seizure is expected to occur, the period between the alarm and the beginning of the SOP is the SPH^16^. A seizure prediction alarm is considered successful if a seizure occurs after the SPH, and within the SOP. The SPH is 0 in some studies^10, 17, 18^, and ranges from 10 s to 4 h in other studies^8, 9, 19^. SOP also varies from 5 min to 1 h in several studies^8^^,^^20^^,^^21^^,^^22^. The SPH mentioned in Park et al.^23^ is actually the SOP definition used in this study, which easily leads to misunderstandings and confusion. Furthermore, not all seizures can be used to collect preictal samples; the study of leading seizures has a much higher value for possible clinical intervention^24^. However, the definition of leading seizure has been entirely overlooked in some studies^8, 10, 18^, and even in those studies that consider this concept, the value of seizure-free time (T) varied from 30 min to 4 h^16^^,^^17^^,^^19^. Under the various choices for T, SOP, and SPH, the generated sample set for training will be completely different even with the same EEG database. Additional details are discussed in "Materials and methods" section.

For model training and evaluation, the sample set should be split into training, validation, and testing sets. A variety of different approaches are used for this task. Tsiouris et al.^25^ shuffled all samples and then used a stratified 10-fold cross-validation to evaluate the prediction performance. Zhang et al.^26^ separated the total dataset into training set and testing set according to the ratio of 8:2. The leave-one-out cross-validation method was used in these two studies^16^^,^^21^. However, the number of available seizures are not equal due to different definitions of leading seizures, which result in a different number of cross-validation experiments. These validation methods are so different that the reliability of the model cannot be guaranteed.

Furthermore, the main aim of seizure prediction research is to improve the patients’ quality of life; thus efficient hardware implementation is very important^11^^,^^27^. However, most research involves complex algorithms that are time-consuming and unfavorable for practical application.

We propose in this work a processing procedure as a reference for training reliably seizure prediction algorithms and facilitating fair benchmarking of successive prediction methods. We investigate the SOP, SPH and T selections for providing trustworthy labels for training and validation. A one-dimensional CNN with Bi-LSTM network is proposed and evaluated on CHB-MIT database shows that it could help improve prediction performance, and the demonstrated power and cost efficiency are advantageous for implant devices.

The remaining sections of this paper are organized as follows: "Materials and methods" section introduces the dataset, data processing method and proposed model structure. "Results" section gives the evaluation results of the proposed model and the comparison results between this work and other works. Several issues are discussed based on the experimental results in "Discussion" section, which greatly influence model performance, including the different choice of sampling parameters and dataset partition methods. "Conclusion" section concludes all the contributions of this work.

## Materials and methods

### Sampling parameters

To clarify the standards for sampling, several important concepts must be defined. As seizures often occur in clusters, the interval between two clusters is defined as the seizure-free time T; the first seizure in each cluster is called a leading seizure. Chen et al.^24^ claimed that T should be based on an analysis of natural seizure clusters. In particular, they recommended listing all of the seizures in chronological order and observing how they naturally cluster, and then recording the longest duration among all natural clusters as the value of T. However, most existing EEG recordings in the public dataset are too short to observe the natural seizure clusters. The value of T can only be chosen as large as possible to obtain more true leading seizures. While the larger the value of T, the less positive samples can be obtained. Considering the trade-off between the number of positive samples and obtaining true leading seizures, T = 4 h is chosen in our study; it is also the most commonly used value in related studies^19^^,^^23^.

SOP and SPH are two important parameters to locate the preictal state accurately on EEG signals. However, the SPH and SOP are often clinically unknown^19^ and are usually chosen based on assumptions, which are significantly different between studies. If SPH is 0^10, 17, 18^, then in the worst situation, there is no time left for patients to prepare in advance for relevant protective measures. In Shiao et al.^19^, the SPH and SOP are 4 h and 1 h, respectively; this period is too long, as even if the model gives a correct alarm, the earliest seizure may occur 4 h later, and the latest is 5 h later; this cause patients to experience premature anxiety. Thus, suitable values for the SOP and SPH not only need to allow the patients to have enough time to take protective measures (i.e., the SPH should be greater than 0) but also to ensure enough preictal samples to train the model (the SOP should be appropriately longer); furthermore, there is a need to avoid unnecessary stress for patients (i.e., neither the SOP nor SPH should be excessively long). According to this analysis and most of the previous works, the sampling parameters used in this work are presented in Table 1. The SPH and SOP are 5 and 30 min respectively, to locate the preictal segments on the long-term EEG recordings. The interictal state was considered as at least 2 h before or after a seizure, to ensure that the segment was far enough away from the preictal period^16^^,^^21^. These segments are further divided into smaller fragments by moving window method to generate the sample set.

**Table 1:** Sampling parameters.

SPH	SOP	T
5 min	30 min	4 h

### Sample set partition and model evaluation

For the purpose of training and testing the model, the sample set should be split into training, validation, and testing sets. For other machine learning problems, the most common method is to divide the dataset according to such as 8:1:1 or other proper ratios or use K-fold cross-validation. But seizure prediction is a time series problem; our purpose is to predict the things that may happen in the future based on present data; the testing set must be invisible throughout the training process. Thus, leave-one-out cross-validation method is used in this work to simulate the clinical situation in real-life. Namely, suppose a patient has N seizures in a dataset. In that case, the preictal samples of the N-1 seizures are used as the training set, and the remaining sample is considered as an impending seizure for evaluating the model performance; this process is repeated N times. We consider that if samples come from the same preictal segment, they have some identical features, leave-one-out cross-validation method could guarantee that the characteristics of testing set will not leak into the training set, thereby ensuring the model’s reliability.

### Dataset

The CHB-MIT EEG dataset was used in this study. This dataset was collected at the Children’s Hospital Boston, consisting of EEG recordings from pediatric subjects with intractable seizures, which were collected by surface electrodes on patients’ scalps. The recordings are achieved using 23 channels and a sampling rate of 256 Hz. The recordings, grouped into 23 cases, were collected from 22 subjects (5 males, ages 3–22; and 17 females, ages 1.5–19)^28^. The data collection followed a protocol approved by the Ethics Committee on Clinical Investigations as the Beth Israel Deaconess Medical Center and the Massachusetts Institution of Technology. All methods were performed in accordance with the relevant guidelines and regulations. Informed written patient consent was also obtained.

### Proposed model

The EEG sample was a matrix with time on the horizontal axis and channels on the vertical axis. Almost all related works treat all channels as a whole, i.e., they feed the entire matrix into the CNN model as the input. Inspired by univariate features in machine learning-based prediction algorithms, we argued that a CNN could also extract features in a single-channel fashion, then designed our CNN model using one-dimensional convolution. The features extracted by CNN need to be fed into a classifier to get the prediction result. We compared two classifier structures, one using the fully connected layers as the classifier directly; the other is to add a Bi-LSTM network between the CNN and the fully connected layers. Bi-LSTM network^29^ is a kind of RNN, which is an improved version of traditional LSTM, has a better feature extraction ability in temporal dimension, and is widely used to process time series data^21^^,^^30^. Through experiments, we found the model’s performance was improved with Bi-LSTM; the comparison results are presented in Table 2.

**Table 2:** Comparison of model structures.

Model structure	Accuracy	Sensitivity	Specificity
CNN + Bi-LSTM + FC	0.776	0.827	0.724
CNN + FC	0.733	0.774	0.691

**Figure 2:**
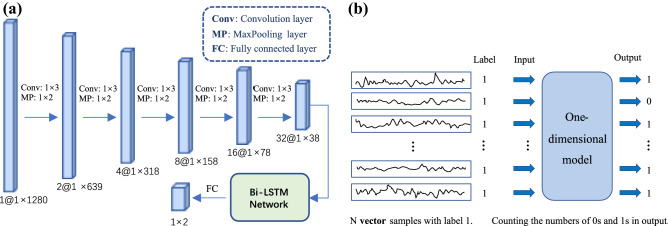
(**a**) One-dimension CNN with a Bi-LSTM network. Conv and MP refers to Convolutional layer and MaxPooling, the following 1 $\times$ 3 and 1 $\times$ 2 indicate the kernel size, the stride of which is 1 and 2, respectively. The Bi-LSTM network has only one recurrent layer, and the number of features in the hidden state is 10. FC refers to a fully connected layer followed by a dropout (rate = 0.5) and a SoftMax function. (**b**) The EEG signals are firstly divided by channels, then voting method in ensemble learning is used to get the final result in the inference stage.

The structure of the proposed model is shown in Fig. 2a. There were five one-dimensional convolutional layers in our proposed model; each layer was followed by batch normalization and a ReLU activation function. The kernel sizes of the convolutional layer and max-pooling layer were 1 and 2, respectively. The Bi-LSTM network had only one recurrent layer, and the number of features in the hidden state was 10. A fully connected layer with a dropout rate of 0.5 and SoftMax function is included in the final classifier.

As a single channel of the signal contains less information than an entire matrix, it was reasonable that the model’s accuracy would decrease when each vector sample was directly classified. However, during the same period, with N channels of EEG signals, using the idea of voting in ensemble learning in the evaluation stage could improve the accuracy. As shown in Fig. 2b, one matrix sample was divided into N vector samples, where N was the number of channels, and the label of each vector was the same as in the original matrix; then, the vector samples were used as the model’s input. The model calculated the categories of N channels separately, and then counted the numbers of 0s and 1s. When the number of positive samples exceeded a set threshold (e.g., N/2), the current EEG sample was considered as positive; otherwise, it was negative.

By splitting EEG signals into individual channels for training and inference, one-dimensional convolutional and max-pooling layers can be applied to design the model, significantly reducing the number of parameters and related computations compared with its two-dimensional counterparts. Moreover, there is no data pre-processing in this work which further reduces the computation burden. For low-power consumption processing, our proposed one-dimensional CNN model will be convenient for hardware implementation.

## Results

In order to evaluate and compare our proposed model, we used the public CHB-MIT database^28^ of EEG signals. It contains 23 epileptic patients’ long-duration recordings as collected by surface electrodes on the patients’ scalps. The recordings are achieved using 23 channels and a sampling rate of 256 Hz.

The proposed model is compared with five state-of-the-art seizure prediction algorithms^16^^,^^21^^,^^26^^,^^31^^,^^32^ that were previously trained and evaluated with CHB-MIT database. Some algorithms^25^^,^^33^^,^^34^ were excluded for comparison because of the complex feature engineering process is unfriendly for implant devices.

For a fair comparison, all models are trained and evaluated with the CHB-MIT database, the T, SOP and SPH was 4 h, 30 min, and 5 min, respectively; the interictal state was 2 h before or after a seizure. Different sampling lengths and feature extraction methods were performed according to the corresponding descriptions in their works. The studies presented in the studies^21^^,^^32^ and our proposed approach used a 5-s moving window with no overlap for sampling; the window lengths in^16^^,^^26^^,^^31^ were 30 s (8 s overlap), 8 s (2 s overlap), and 20 s (5 s overlap), respectively. Truong et al.^16^ converted the EEG samples into a two-dimensional image by short-time Fourier transform. The Pearson correlation coefficient (PCC) for each pair of channels was required to generate the correlation matrix^26^. Raw EEG signals were used in these studies^21^^,^^31^^,^^32^, and our proposed model. In the following, we compare the different models in terms of prediction performance and implementation feasibility for implant devices.

With 4 h seizure-free time T, the available seizures (true leading seizures) of each patient are decreased, and the leave-one-out validation method requires at least three seizures for cross-validation. Combining these two conditions, only subjects 1, 6, 8, 9, 10, 18, 22 have at least three leading seizures; the experimental results on these subjects are presented in Table 3. The second column indicates which leading seizure is used as the testing set, the last row shows the average accuracy, sensitivity and specificity of each model. Our model achieves the highest accuracy (80.0%) and sensitivity (87.7%), significantly higher than that of the second-ranked, which are 75.3% and 81.5%, respectively. Although the 72.3% specificity is not the highest, but it ranks second among all the models, and the other two indicators of our model are much higher than presented in Xu et al.^31^. As a result, with fewer parameters and less computing resources requirement, the proposed model achieves the highest accuracy and sensitivity.

**Table 3:** Evaluation results of using leave-one-out cross-validation method to test different models.

Subject/ Seizures		Daoud et al.^21^			Truong et al.^16^			Zhang et al.^26^			Xu et al.^31^			Lawhern et al.^32^			This work		
		ACC^1^	TPR^2^	TNR^3^	ACC	TPR	TNR	ACC	TPR	TNR	ACC	TPR	TNR	ACC	TPR	TNR	ACC	TPR	TNR
1	1	0.974	0.992	0.955	0.975	0.988	0.963	0.916	0.957	0.876	0.992	0.992	0.992	0.968	0.975	0.961	0.922	0.855	0.989
	3	0.945	1.000	0.891	0.894	1.000	0.788	0.939	0.997	0.882	0.885	0.991	0.778	0.910	1.000	0.821	0.972	1.000	0.944
	7	0.902	0.812	0.992	0.875	0.800	0.950	0.714	0.746	0.682	0.631	0.602	0.661	0.815	0.756	0.874	0.801	0.798	0.804
6	1	0.724	0.651	0.796	0.805	0.828	0.781	0.712	0.746	0.678	0.702	0.606	0.798	0.734	0.609	0.859	0.870	0.951	0.789
	4	0.282	0.121	0.444	0.310	0.570	0.051	0.318	0.037	0.598	0.393	0.077	0.709	0.456	0.427	0.486	0.374	0.126	0.621
	6	0.705	0.760	0.649	0.722	0.790	0.654	0.672	0.692	0.652	0.676	0.504	0.849	0.742	0.774	0.710	0.774	0.972	0.577
	8	0.684	0.699	0.669	0.829	0.949	0.709	0.720	0.861	0.578	0.504	0.966	0.043	0.718	0.772	0.663	0.810	0.983	0.638
	9	0.779	0.680	0.877	0.926	0.864	0.988	0.838	0.896	0.779	0.651	0.403	0.899	0.815	0.716	0.914	0.983	0.969	0.997
	10	0.603	0.721	0.485	0.599	0.605	0.593	0.714	0.926	0.502	0.660	0.882	0.437	0.627	0.721	0.532	0.655	0.961	0.348
8	1	0.840	0.705	0.975	0.877	0.802	0.951	0.992	0.983	1.000	0.958	1.000	0.916	0.865	0.733	0.997	0.996	0.992	1.000
	3	0.834	0.710	0.958	0.994	0.988	1.000	0.975	0.990	0.960	0.962	1.000	0.924	0.721	0.632	0.811	0.939	0.942	0.936
	5	0.544	0.890	0.197	0.488	0.038	0.938	0.740	0.970	0.103	0.742	0.923	0.283	0.558	0.874	0.242	0.492	0.421	0.562
9	1	0.745	0.889	0.602	0.728	0.741	0.716	0.644	0.639	0.649	0.605	0.319	0.891	0.756	0.894	0.618	0.813	0.877	0.749
	2	0.625	0.549	0.702	0.728	0.852	0.605	0.734	0.612	0.856	0.697	0.513	0.882	0.618	0.451	0.786	0.774	0.749	0.799
	4	0.706	0.763	0.649	0.741	0.864	0.617	0.554	0.258	0.849	0.693	0.773	0.613	0.709	0.694	0.724	0.713	0.816	0.610
10	1	0.485	0.538	0.432	0.574	0.691	0.457	0.547	0.860	0.234	0.429	0.807	0.050	0.552	0.240	0.864	0.532	0.755	0.309
	2	0.735	0.747	0.724	0.809	0.815	0.802	0.935	0.943	0.926	0.731	0.555	0.908	0.712	0.571	0.852	0.915	0.986	0.844
	3	0.648	0.710	0.585	0.488	0.395	0.580	0.701	0.977	0.425	0.710	0.874	0.546	0.834	0.850	0.819	0.760	0.880	0.641
	4	0.359	0.273	0.446	0.574	0.852	0.296	0.420	0.080	0.759	0.420	0.370	0.471	0.517	0.306	0.727	0.311	0.003	0.618
	6	0.770	0.844	0.696	0.870	0.815	0.926	0.871	0.963	0.779	0.668	0.723	0.613	0.748	0.827	0.669	0.935	0.978	0.891
	7	0.679	0.456	0.902	0.729	0.688	0.771	0.718	0.603	0.832	0.754	0.873	0.634	0.919	0.926	0.912	0.972	0.944	1.000
18	1	0.471	0.373	0.568	0.728	0.827	0.630	0.692	0.445	0.940	0.634	0.571	0.697	0.595	0.688	0.501	0.678	0.727	0.630
	5	0.295	0.120	0.471	0.716	0.556	0.877	0.778	0.773	0.783	0.571	0.941	0.202	0.607	0.652	0.563	0.645	0.925	0.365
	6	0.935	0.871	1.000	0.917	0.833	1.000	0.750	0.846	0.654	0.944	0.889	1.000	0.710	0.516	0.903	1.000	1.000	1.000
22	1	0.670	0.702	0.638	0.796	0.827	0.765	0.804	0.967	0.642	0.794	1.000	0.588	0.689	0.652	0.727	0.804	0.811	0.797
	2	0.412	0.315	0.510	0.488	0.926	0.049	0.455	0.853	0.057	0.466	0.504	0.429	0.478	0.474	0.482	0.552	0.916	0.187
	3	0.783	0.733	0.832	0.869	0.881	0.857	0.940	0.975	0.906	0.881	0.825	0.937	0.817	0.853	0.780	0.955	0.995	0.916
Average		0.672	0.653	0.691	0.743	0.770	0.715	0.733	0.763	0.688	0.695	0.722	0.657	0.711	0.688	**0.733**	**0.776**	**0.827**	0.724

The first two columns refer to the subjects and the number of seizures used as the test set.

^1^Accuracy.

^2^True positive rate (Sensitivity).

^3^True negative rate (Specificity).

Table 4 shows the required number of model parameters and the number of floating-point operations. The number of parameters is related to the required memory for storing the model, therefore affecting the size of the implant devices. Integrated in chips based implantable devices, the on-chip memory must be small, up to 200 kb. In addition to model parameters, the intermediate calculation results also need to be stored in the silicon chip. Thus, models with fewer parameters are more feasible for area-critical implant chips. The parameters of our proposed model and those of the model proposed by Lawhern et al.^32^ are both less than 10 kB, suitable for power budget and memory constrained implantable devices. The number of floating-point operations are directly related to the power consumption of a prediction processor. Achievements published in Daoud et al.^21^ and Xu et al.^31^ require giga-level operation, making them impossible for low-power consumption biochips’ implementation. The studies^16^^,^^26^^,^^32^ require a few hundred mega operations, although proved feasible for low-power microcontroller units platforms^35^, they are only suitable for wearable devices where power consumption is still at a few mW level. The required floating-point operations of our model count only 12.825M, which are more than twentyfold lower than other models, the low-computation requirement gives unique advantages for low-power consumption of implants’ implementation. Our model has the least floating-point operations and requires the least power consumption, and the number of parameters of our model is only slightly more than that of Lawhern et al.^32^, therefore is more suitable for hardware implementation.

**Table 4:** The feature extraction methods, floating-point operations, and parameters of different model.

Models	Feature extraction	Floating-point operations	Parameters
Daoud et al.^21^	Raw data	1.844 G	47.574 k
Truong et al.^16^	STFT^1^	302.442 M	224.066 k
Zhang et al.^26^	PCC^2^	205.144 M	75.010 k
Xu et al.^31^	Raw data	6.739 G	2.040 M
Lawhern et al.^32^	Raw data	504.932 M	2.384 k
This work	Raw data	12.825 M	6.274 k

^1^Short-time Fourier transform.

^2^Pearson correlation coefficient.

## Discussion

In Table 3, when testing seizure 1 of chb08, seizure 1 of chb10, and seizure 2 of chb22, all the models have a high sensitivity, but the accuracy is approximately 50%, and the specificity is close to 0. It indicates that all samples are predicted as preictal stages. The indicators of seizure 4 of chb06 and seizure 4 of chb10 are also abnormal, as the accuracy is much lower than 50%. The performance of a CNN model is often related to three factors: data, model, training process. Among these six models, different feature extraction methods (raw data, STFT and PCC), different model structure design and multiple cross-validations were performed, but all models showed similar abnormalities on the same test set. Thus, we consider that these abnormalities are only related to the training data, which could be explained from two aspects: sampling parameters and sample set partition method.

As discussed in "Materials and methods" section, without knowing the ground truth, the sampling parameters of SOP and SPH are only hypothetical values. The ground truth SOP and SPH can vary greatly among different individuals; even in the same subject, the values can change over time^36^. Hence, the preictal segment and the prototypical physiological preictal signature (the ground truth) cannot match perfectly^37^. All the samples from the preictal segments are labeled as 1 (positive samples), as they are assumed to have similar characteristics^38^. But there can be some samples that without such characteristics are labeled incorrectly due to the mismatch with the ground truth. The greater the number of false samples in the training set, the more likely the model will predict a negative sample as a positive. When such a model is tested on a normal testing set (with no or just a few false samples), it will generate many false positive predictions (negative samples are predicted as positive). This explains why the specificity is lower than sensitivity in seizure 1 of chb08, seizure 1 of chb10, and seizure 2 of chb22. However, if the testing set itself has many false samples, the situation becomes more complex as the low accuracy shown in seizure 4 of chb06 and seizure 4 of chb10. The two sampling parameters SPH and SOP are set as 5 min and 30 min respectively in this work to fit the ideal prediction situation, but these values are not suitable for those abnormal cases.

A grid search method can be used to find the optimal values for the SOP and SPH, but when we increased the SPH to 30 min or longer, we could not obtain any preictal samples from some seizures, owing to missing data.

**Figure 3:**
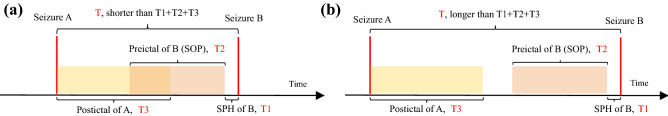
Different time intervals between two seizures: (**a**) There is an overlap between postictal and preictal, (**b**) No overlap between postictal and preictal.

In addition to SOP and SPH, seizure-free time T is another sampling parameter. It is taken into account the distinction between leading and follow-up seizures during sampling. As shown in Fig. 3, the postictal duration of seizure A is T1 and the SOP and SPH of seizure B are T2 and T3, respectively, the time interval between seizure A and B is T. If T is too short and is less than T1+T2+T3, i.e., no distinction is made between leading and follow-up seizures, there will be some overlap between the postictal (of seizure A) and preictal (of seizure B) segments (Fig. 3a), indicating that the preictal segment contains some features of the postictal stage. The postictal stage is a period that measures the time it takes for the patient to return to normal after a seizure occurs, which is much easier to predict than the true preictal segment^24^. Thus, the model may achieve overly optimistic results under this situation. On the contrary, if T is greater than T1+T2+T3 (Fig. 3b), the preictal data is clean, contains no other noise signals or features, which is important to build a reliable prediction model. However, the duration of the postictal stage is unknown as the existing databases do not contain such annotations; we can only make T as large as possible to avoid overlap between preictal and postictal.

**Figure 4:**
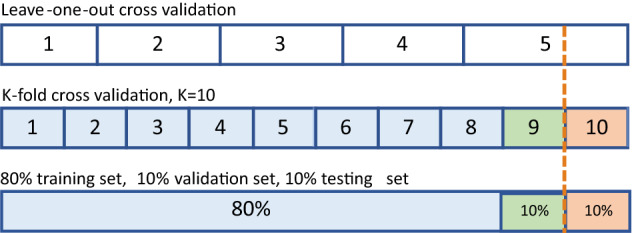
Different dataset partition methods. Assuming the patient has 5 seizures in total, the top frame is the leave-one-out cross-validation, the 5 labeled rectangles correspond to the preictal segment of 5 different seizures. The samples are split as K-fold and 8:1:1 respectively in the following two frames, where K is 10 in this instance.

In addition to the leave-one-out cross-validation method, Fig. 4 shows another two commonly used preictal sample set partition methods. The top frame is assumed to be the preictal sample set of a certain patient whose EEG signals include five seizures in total. The numbers indicate that from which seizure the preictal segment samples were collected. Owing to missing data, the sample sizes of each preictal segment were not equal, as reflected in the different widths of the rectangles in the top frame. The sample set is split into ten parts in the middle frame, one as validation set, one as the testing set, and the remaining eight parts as training set, which is K-fold cross-validation. In the bottom frame, the sample set is split into training, validation and testing set as a ratio of 8:1:1. As mentioned in "Materials and methods" section, the last two dataset partition methods do not take the real-life situation into account but simply divide the sample set mathematically. When predicting an epileptic seizure, all the data of this seizure should be unknown. Thus, the testing set should simulate such a situation, and the training set can only contain historical data. In Fig. 4, the training, validation and testing sets are marked as blue, green and orange, respectively. It can be noted that, except for leave-one-out cross-validation method, the other two methods divide the preictal samples of seizure No. 5 into two parts (dashed lines), namely, the samples from the same seizure (seizure No. 5) exist in both the training and testing sets, which leads to data leakage from testing set to training set. The situation of validation set is similar to the testing set; it could not be guaranteed that the model is not overfitting. Actually, we have observed around 10% performance increase due to the overfitting. Thus, models usually perform not ”so well” when using leave-one-out cross-validation method. According to Hussein et al.^39^, the samples with the same state (preictal or interictal) have similar features, but the features can change over time. This means there are some differences between two preictal segments. In other words, preictal samples have some common features (global features) and some specific features (local features). As such, the model’s performance may not always be good on different cross-validation sets.

A model can easily achieve high accuracy when ignoring the preceding rules; nevertheless, we should focus on the model’s generalization ability instead of simply pursuing high performance on a small dataset. This is why we emphasized that the proposed procedure should be followed. Furthermore, high-quality data and reliable annotation are two critical issues in supervised machine learning and deep learning problems. Although the SPH and SOP are clinically unknown, other prior knowledge may also be helpful for seizure prediction research. For example, by observing the states of the patients, specialist physicians might be able to determine the duration of the postictal state, seizure types, temperatures, and other signs in the patients, along with which seizures belong to the same cluster.

## Conclusion

In this work, we analyzed the effects of T, SOP, SPH on the sample set. We also discussed the different evaluation methods and the corresponding dataset partition methods. All these factors have great impact on the model’s performance. We also proposed a CNN model and a novel single-channel training and inferring method. The model consists of one-dimensional convolutional layers and a Bi-LSTM network, has 6.274 k parameters and require 12.825 M floating-point operations, which is faster and more lightweight than previous models. The proposed model achieves 77.6% accuracy, 82.7% sensitivity, and 72.4% specificity on public dataset CHB-MIT, outperforms the state-of-the-art works.

## Data Availability

The EEG data used in this work is from a public database (CHB-MIT scalp EEG database), which could be accessed and downloaded via https://archive.physionet.org/physiobank/database/chbmit/.
